# Abscence of the Ductus Communis Choledochus

**Published:** 1870-02

**Authors:** I. N. Danforth

**Affiliations:** Chicago


ARTICLE VII. — *Absence of the Ductus Communis Choledochus.* By I. N. DANFORTH, M.D., Chicago.

SEVERAL years ago, while in practice in a New England town, I was employed to attend a woman in her third confinement. After an easy and natural labor of some four hours duration, she was delivered of a well-developed boy, weighing a little over nine pounds. For the twenty-four hours after birth, the child betrayed no abnormal symptoms. At my next visit, however (about thirty hours after delivery), I noticed an icteric appearance of the countenance, and upon closer inspection, a well-marked yellow tinge of the whole surface was discovered. The nurse informed me that the discharges from the bowels were "almost like clay," and that the child had frequent attacks of vomiting. During the afternoon of the succeeding day (nearly sixty hours after birth), the skin had assumed an intensely yellow color, and the stomach was in a very irritable condition; at the same time, there were evident indications of hunger — the child would seize the breast with avidity, but would almost immediately forsake it in disgust. The symptoms continued to grow worse; the color of the skin to changed to a brownish-yellow or bronze; the irritability of the stomach increased? convulsions supervened, and, in about twelve hours after my second visit, or seventy-two hours after birth, the child died in a state of profound coma.

*Sectio cadaveris.* — The tissues throughout the body were stained intensely yellow. The heart, lungs, stomach, intestines, spleen, and kidneys were all perfectly developed and healthy. The liver was swollen and enlarged, and this was evidently due to distension of the biliary ducts, as, upon cutting into it, an unusual amount of very thick bile oozed from the cut surfaces. The gall-bladder occupied its normal position, and was enormously distended with bile of about the consistency of syrup. The cystic and hepatic ducts presented nothing unusual, except that they were very much enlarged, a point I shall allude to again; they united at the usual place to form the common duct. The ductus communis choledochus — also very greatly distended — was about three-quarters of an inch long; it then terminated abruptly, in a very blunt club-shaped extremity, without reaching the intestinal canal at all.

Of course, the symptoms and cause of death were at once solved, upon making this remarkable discovery. The end of the duct presented a curious appearance; one might almost imagine that it had been suddenly cut off with a sharp instrument. Scarcely could a round billet of wood be sawn off with more accuracy and neatness.

I have already mentioned the great distension and enlargement of all the ducts. I could, without difficulty, pass my finger into the gall-bladder, through the cystic duct. Upon this point, Rokitansky remarks (Path. Anat. Vol. II., p. 124): "The gall-ducts are capable of extensive distension."

I have not been able to find any such case upon record, although I have carefully examined such authorities as came within my reach. Jones and Sieveking remark (Path. Anat., p. 525): "The gall-badder is sometimes wanting — in animals it has been found double; its shape may be variously deformed; its duct, as well as the common duct, may probably be imperforate." It is quite possible, however, that analogous cases may be found in medical journals of this, or other countries, which have escaped my notice. But such cases are sufficiently rare to warrant the publication of all which do unfortunately happen.

